# Lung cancer patients with chronic obstructive pulmonary disease benefit from anti-PD-1/PD-L1 therapy

**DOI:** 10.3389/fimmu.2022.1038715

**Published:** 2022-12-01

**Authors:** Mao Lin, Zongyao Huang, Yingfu Chen, Hongtao Xiao, Ting Wang

**Affiliations:** ^1^ Department of Pharmacy, Sichuan Cancer Hospital and Institute, Sichuan Cancer Center, School of Medicine, University of Electronic Science and Technology of China, Chengdu, Sichuan, China; ^2^ Department of Pathology, Sichuan Cancer Hospital and Institute, Sichuan Cancer Center, School of Medicine, University of Electronic Science and Technology of China, Chengdu, Sichuan, China; ^3^ Department of Pharmacy, Clinical Medical College and Affiliated Hospital of Chengdu University, Chengdu, Sichuan, China; ^4^ Department of Clinical Research, Sichuan Cancer Hospital and Institute, Sichuan Cancer Center, School of Medicine, University of Electronic Science and Technology of China, Chengdu, Sichuan, China

**Keywords:** chronic obstructive pulmonary disease (COPD), immune checkpoint inhibitors (ICIs), PD-1/PD-L1, lung cancer (LC), tumor immune microenvironment (TIME)

## Abstract

Lung cancer (LC) and chronic obstructive pulmonary disease (COPD) are two of the most fatal respiratory diseases, seriously threatening human health and imposing a heavy burden on families and society. Although COPD is a significant independent risk factor for LC, it is still unclear how COPD affects the prognosis of LC patients, especially when LC patients with COPD receive immunotherapy. With the development of immune checkpoint inhibition (ICI) therapy, an increasing number of inhibitors of programmed cell death-1 (PD-1) and PD-1 ligand (PD-L1) have been applied to the treatment of LC. Recent studies suggest that LC patients with COPD may benefit more from immunotherapy. In this review, we systematically summarized the outcomes of LC patients with COPD after anti-PD-1/PD-L1 treatment and discussed the tumor immune microenvironment (TIME) regulated by COPD in LC immunotherapy, which provides novel insights for the clinical treatment of LC patients with COPD.

## 1 Introduction

Chronic obstructive pulmonary disease (COPD) is a common and fatal respiratory disease. It is a heterogeneous syndrome consisting of emphysema, chronic bronchitis, and small airway disease, and it affects approximately 251 million people worldwide (1). COPD is a devastating lung disease that is characterized by progressive airflow restriction and is associated with the abnormal inflammatory response of the lung to noxious particles or gases (1, 2). The World Health Organization (WHO) predicts that by 2030, if effective measures are not taken, such as reducing smoking and air pollutants, COPD will become the third leading cause of death globally (3). In addition, during the same period, the number of lung cancer (LC) deaths will rise to 10 million annually, accounting for nearly one-fifth of all cancer deaths worldwide (4, 5).

LC and COPD share common risk factors (such as smoking and other environmental factors), and the pathogenesis of them is the same. As early as the 1980s and 1990s, researchers observed that chronic lung disease might participate in LC progression and explored the relationship between COPD and LC (6, 7). In 1986, Skillrud et al. first proposed that COPD could serve as an important independent risk factor for LC (7). Previous studies reported that respiratory symptoms such as coughing, expectoration, shortness of breath, and chest tightness, as diagnostic criteria for COPD, had a negative impact on the prognosis of LC patients (8–10). However, the application of immune checkpoint inhibitors (ICIs), especially PD-1/PD-L1 inhibitors, in advanced LC, along with evidence of the imbalance of immune checkpoint protein (PD-1 and PD-L1) expression and changes in the immune microenvironment in COPD patients (8, 11, 12), revealed that COPD-related LC may respond better to immunotherapy (8, 13). Therefore, we systematically summarized the outcomes of LC patients with COPD after PD-1/PD-L1 inhibitor therapy and explored the impact of the tumor immune microenvironment (TIME) regulated by COPD on LC immunotherapy.

## 2 Current advances in anti-PD-1/PD-L1 therapy in LC patients with COPD

**Figure 1:**
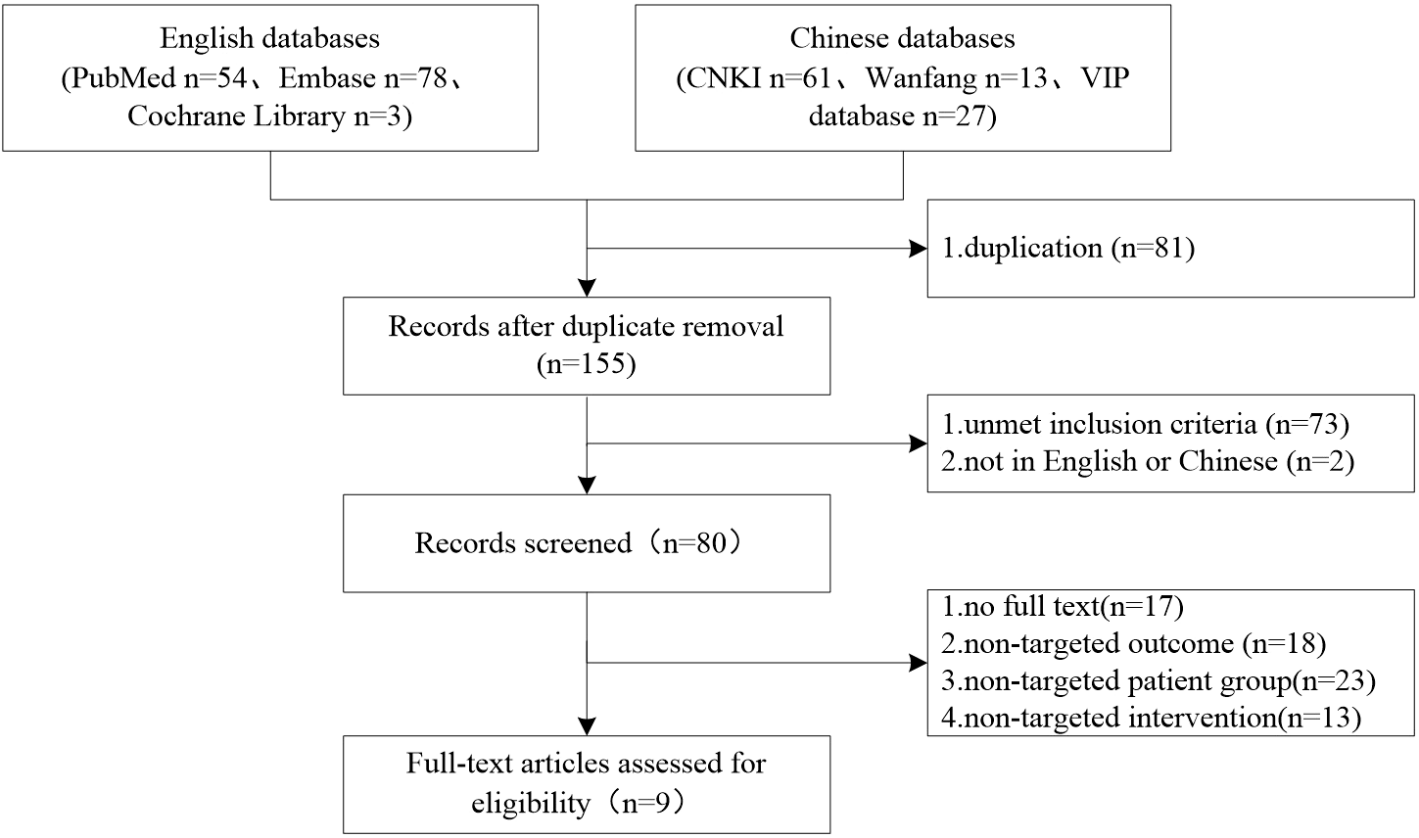
Flowchart of the literature search for ICI therapy in LC patients with COPD.

To understand the current status of immunotherapy in LC patients with COPD, we conducted a rapid systematic review. Inclusion criteria: 1) population: LC patients with COPD at any disease stage; 2) interventions: ICI immunotherapy, with no restrictions on drug regimen and treatment lines; 3) outcomes: the overall survival (OS), progression-free survival (PFS), objective response rate (ORR) and the impact on pulmonary function; 4) research type: randomized controlled trials (RCTs) and cohort studies. Exclusion criteria: 1) duplicate publications; 2) languages other than Chinese or English; 3) conference abstracts or studies without data; 4) literature with inconsistent research purposes; 5) unavailable studies. Then, all English databases (PubMed, Embase, Cochrane Library) and Chinese databases (CNKI, VIP, WanFang databases) from the inception of the database to 8^th^ July 2022 were searched. The search terms were (chronic obstructive pulmonary disease OR COPD OR ventilatory defect OR emphysema) AND (immune checkpoint inhibitor OR pembrolizumab OR atezolizumab OR nivolumab OR durvalumab OR carrelizumab OR toripalimab OR sintilimab OR tislelizumab OR immunity therapy OR PD-1 OR PD-L1) AND (Lung cancer). The search formula of PubMed is shown in **Table 1**. Finally, a total of 236 articles (135 in English and 101 in Chinese) were retrieved, but only 9 studies met the inclusion criteria and were included. The screening process is shown in **Figure 1**.

**Table 1:** The search formula of PubMed.

Search number	Query
#1	Search: chronic obstructive pulmonary disease[MeSH Terms]
#2	Search: (((chronic obstructive pulmonary disease[Title/Abstract]) OR (COPD[Title/Abstract])) OR (ventilatory defect[Title/Abstract])) OR (emphysema[Title/Abstract])
#3	#1 OR #2
#4	Search: immune checkpoint inhibitor[MeSH Terms]
#5	Search: (((((((((((immune checkpoint inhibitor[Title/Abstract]) OR (pembrolizumab[Title/Abstract])) OR (atezolizumab[Title/Abstract])) OR (nivolumab[Title/Abstract])) OR (durvalumab[Title/Abstract])) OR (carrelizumab[Title/Abstract])) OR (toripalimab[Title/Abstract])) OR (sintilimab[Title/Abstract])) OR (tislelizumab[Title/Abstract])) OR (immunity therapy[Title/Abstract])) OR (PD-1[Title/Abstract])) OR (PD-L1[Title/Abstract])
#6	#4 OR #5
#7	Search: (Lung cancer[MeSH Terms]) OR (Lung cancer[Title/Abstract])
#8	#3 AND #6 AND #7

In total, 9 relevant studies were included, 2 from China (8, 14), 4 from Japan (15–18), 1 from the United States (19), 1 from South Korea (20) and 1 from France (21). All studies were cohort studies (prospective 2 and retrospective 7). There were 1044 patients with stage III-IV NSCLC (COPD 432 and non-COPD 612). The ICIs used in the studies included nivolumab, pembrolizumab, atezolizumab, avelumab, camrelizumab, tislelizumab and PD-1/PD-L1 inhibitors.

Seven studies reported OS or median OS (COPD vs. non-COPD, respectively: nonreached vs. 510 days, P<0.05; COPD better, P = 0.0126; 19.5 vs. 11.6 months, P=0.03; 20.6 vs. 10.8 months, P = 0.092; 359 vs. 145 days, P = 0.0350; COPD better, P=0.003; COPD better, P=0.2), and these studies also revealed longer PFS or median PFS in the COPD-LC group (COPD vs. non-COPD: 316 vs. 186 days, P=0.018; COPD better, P = 0.0407; 6.6 vs. 2.7 months, P<0.001; 6.5 vs. 2.3 months, P<0.01; 154 vs. 44 days, P = 0.0491; COPD better, P=0.003; COPD better, P=0.04). In addition, 5 studies reported ORR (COPD vs. non-COPD: 52.63% vs. 90.91%, P<0.05; 75.0% vs. 53.7%, P = 0.0586; 36.4% vs. 20.8%, P = 0.0167; 32.4% vs. 15.9%, P=0.022; 38.2% vs. 20.5%, P=0.028). The 2 other studies showed that FeNO levels, FVC and FEV1 were significantly increased in the COPD-LC group after immunotherapy (P<0.05), while there was no significant change in the LC group (P>0.05).

Overall, these studies suggested that COPD is not a risk factor for LC patients receiving immunotherapy. Conversly, LC patients with COPD may benefit more than non-COPD LC patients, with better PFS, OS and ORR, as well as increased FeNO levels and improved lung function based on FVC and FEV1; the details are specified in **Table 2**.

**Table 2:** Main characteristics of studies in systematic reviews.

Study ID	Country	Type of Study	Disease Stage	Drug	COPD vs non-COPD		
					Population	Age(years)	Outcome
Jiebai Zhou, 2021 (8)	China	retrospective cohort study	Advanced-stage NSCLC (n=156)	PD-1/PD-L1 inhibitors	65 vs. 91	/	median OS: non-reached vs. 510 days, P<0.05; median PFS: 316 vs. 186 days, HR = 0.56, 95% CI (0.33,0.96), P=0.018
Huo Shufen, 2022 (14)	China	prospective cohort study	Recurrence、IIIA-IV NSCLC (n=30)	Pembrolizumab/Nivolumab/Carrelizumab/Tislelizumab	19 vs. 11	All: 67.2 ± 9.8	ORR: 52.63% vs. 90.91%, P<0.05; FeNO levels: 26.08 (18.32, 32.91) vs. 21.10 (15.58, 24.84), P<0.05; FVC: 2.98 ± 0.35 vs. 2.62 ± 0.38, P<0.05; FEV1: 1.99 (1.65, 2.18) vs. 1.98 (1.60, 2.20), P<0.05
Yuzo Suzuki, 2019 (15)	Japan	prospective cohort study	IIIB, IV, or unresectable stage IIIA NSCLC (n=95)	Pembrolizumab	41 vs. 54	69 (67–73) vs. 69 (63–75)	ORR: 75.0% vs. 53.7%, P = 0.0586; FeNO levels: COPD increased (P =0.0242), non-COPD didn’t (P>0.05) FVC/FEV1: COPD increased (P<0.05), non-COPD didn’t (P>0.05)
Shinkichi Takamori, 2020 (16)	Japan	retrospective cohort study	Advanced or recurrent NSCLC (n=257)	Nivolumab/Pembrolizumab/Atezolizumab	57 vs. 200	61.8 vs.65.0	OS: COPD better, P = 0.0126; PFS: COPD better, P = 0.0407; ORR: 36.4% vs. 20.8%, P = 0.0167
Yusuke Takayama, 2021 (17)	Japan	retrospective cohort study	Advanced-stage NSCLC (n=153)	Nivolumab/Pembrolizumab/Atezolizumab	71 vs. 82	68.0 ± 9.5 vs. 68.0 ± 10.3	median OS: 19.5 vs. 11.6 months, HR=0.58, 95% CI (0.36, 0.94), P=0.03; median PFS: 6.6 vs. 2.7 months, HR=0.47,95% CI (0.32,0.69), P<0.001; ORR: 32.4% vs. 15.9%, P=0.022
Yoshimi Noda, 2022 (18)	Japan	retrospective cohort study	Advanced-stage NSCLC (n=56)	PD-1/PD-L1 inhibitors	41 vs. 15	70 (66–74) vs. 72 (64–75)	OS: 20.6 vs. 10.8 months, P = 0.092; PFS: 6.5 vs. 2.3 months, P < 0.01
Nicholas M. Mark, 2018 (19)	USA	retrospective cohort study	III-IV NSCLC (n=125)	Pembrolizumab/Nivolumab/Atezolizumab/Avelumab	60 vs. 65	68.8 ± 7.0 vs. 64.3 ± 10.1	OS: 359 vs. 145 days, P = 0.0350; PFS: 154 vs. 44 days, P = 0.0491
Sun Hye Shin, 2019 (20)	Korea	retrospective cohort study	Advanced-stage NSCLC (n=133)	Pembrolizumab	59 vs. 74	65.3 ± 8.0 vs. 61.0 ± 10.2	OS: COPD better, HR=0.45, 95%CI (0.26,0.78), P=0.003; PFS: COPD better, HR=0.50; 95%CI (0.31,0.79), P=0.003; ORR: 38.2% vs. 20.5%, P=0.028
Jérôme Biton, 2018 (21)	France	retrospective cohort study	Advanced-stage NSCLC (n=39)	Nivolumab	19 vs. 20	64 ± 9 vs. 61 ± 12	OS: COPD better, P=0.2 PFS: COPD better, P=0.04

OS, Overall survival; PFS, progression-free survival; ORR, objective response rate; FEV1, forced expiratory volume in one second; FVC, forced vital capacity.

## 3 Potential mechanisms for LC patients with COPD benefiting from PD-1/PD-L1 inhibitor treatment

### 3.1 Interactions between PD-1/PD-L1 inhibitors and the tumor immune microenvironment (TIME)

#### 3.1.1 Mechanisms of action of PD-1/PD-L1 inhibitors

Effective antitumor immunotherapy mainly relies on the modulation of the tumor microenvironment and restoration of the T-cell response. The activation of T cells requires two signals: the first signal is antigen recognition, which comes from the binding of T-cell antigen receptor (TCR) to cognate antigen presented in the context of major histocompatibility complex (MHC) on the surface of antigen-presenting cells (APCs), and the second signal is provided by the interactions between costimulatory molecules, which are also called ‘immune checkpoint’ molecules (22). The second signals are divided into two types: costimulatory signals (classical pathways include B7.1/B7.2/B7H2-CD28, CD137 L-CD137, CD70-CD27, CD40-CD40 L) and coinhibitory signals (classical pathways include B7H1/B7DC-PD1, B7.1/B7.2/B7H2-CTLA4, HVEM-BTLA), which perform positive and negative regulatory functions, respectively (23–26).

The PD-1 receptor is expressed on the surface of T cells and primary B cells and plays an important role in the regulation of cell differentiation and apoptosis. PD-1 has two ligands, PD-L1 (B7-H1) and PD-L2 (B7-DC) (27). PD-L1 protein is widely expressed in activated T cells, B cells and macrophages and interacts with the receptor PD-1 on T cells to inhibit the activation of T cells and cause apoptosis in these cells, thus exerting suppressive effects on the immune response in various cancer types. Furthermore, the tumor microenvironment can also induce the expression of PD-L1, which induces the apoptosis of antitumor T cells and contributes to the occurrence and growth of tumors (23, 28, 29).

Under normal circumstances, APCs such as macrophages and dendritic cells can capture foreign pathogens or antigens, then process them to bind with MHC molecules, and present them outside the cells for T cells to recognize through the TCR (23, 24). In addition to MHC-TCR contact, costimulatory signals such as CD80/CD86-CD28 activate effective and sufficient T cells and initiate T-cell immune responses (25, 29). To avoid excessive T-cell activation caused by continuous antigen stimulation, coinhibitory molecules such as PD-1 and PD-L1 are transmitted in T cells, thus reducing the proliferation or apoptosis of T cells and avoiding excessive immune activation (22), which could prevent T-cell killing of tumor cells. However, it is necessary to stimulate the immune response of T cells in cancer therapy. Consequently, ICIs (PD-1/L1 inhibitors) can block the combination of PD-1/PD-L1 and negative regulatory signaling, recover the functional activity of T cells, and thus enhance the immune response against tumor cells.

#### 3.1.2 TIME characteristics may be a vital determinant of the efficacy of ICI treatment

The tumor immune microenvironment (TIME) is a complex and dynamic ecosystem that consists of many different cell types, including tumor cells, immune cells and other supporting cells (e.g., fibroblasts, stromal cells and endothelial cells) (30). Circulating immune cells can be recruited by chemokines produced by tumor cells, fibroblasts or inflammatory cells and then migrate to the tumor site through the transendothelial process. As a result of the abnormal components and functions of immune cells in the TIME, tumor cells escape immunity and become drug resistant and metastasize. Among the multiple cell populations in the TIME, people can often find cells associated with acute inflammation (including neutrophils, basophils, and eosinophils), cells associated with innate immunity (including macrophages, NK cells, and DCs), and cells derived from adaptive immune responses (including CD8+ T cells, Th1-/Th2 cells, and B cells) (30, 31). Collectively, the TIME formed by the interaction between immune cells affects the invasion and metastasis of tumor cells, which can further modulate the progression of cancer (32).

**Figure 2:**
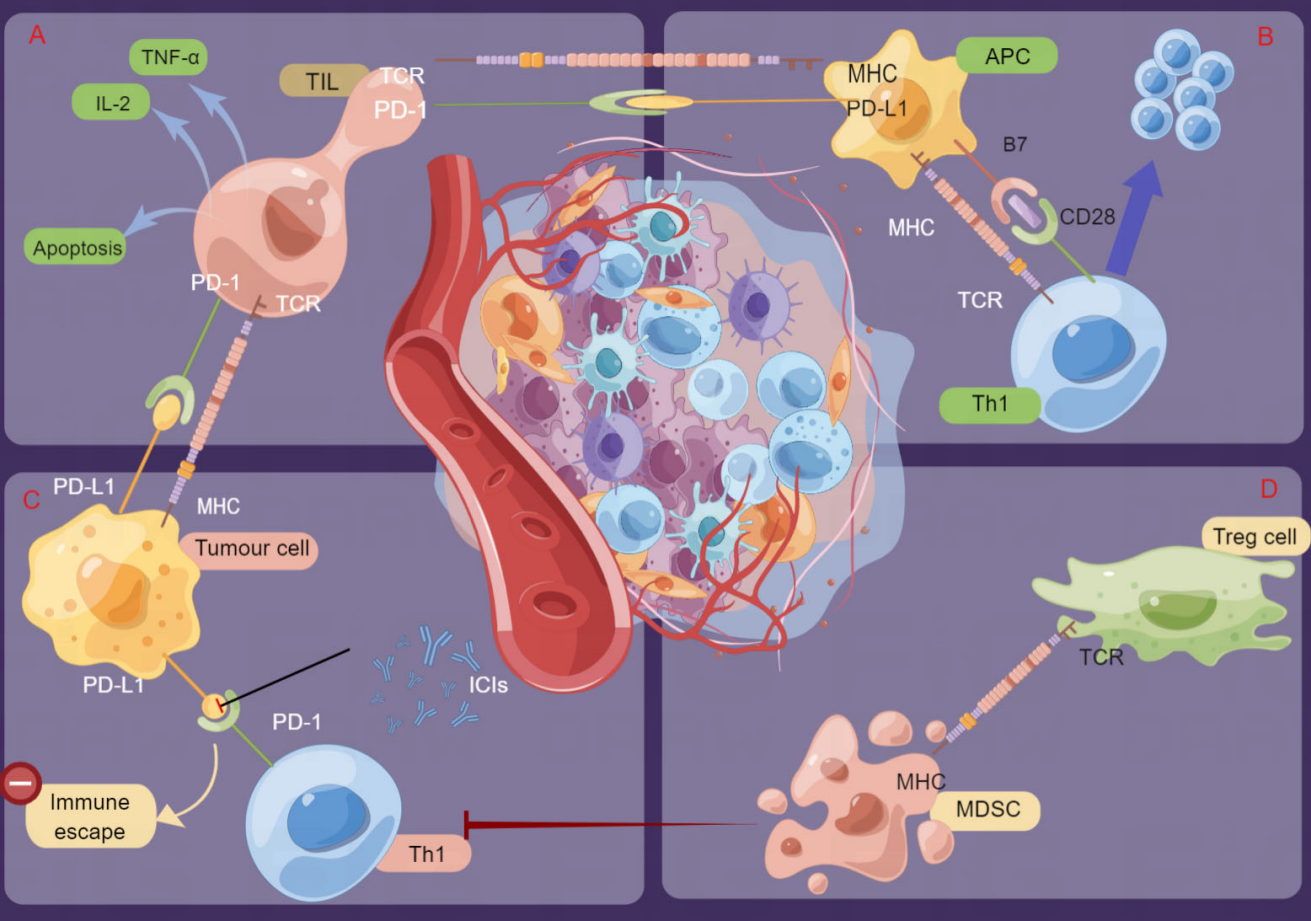
Schematic presentation of the mechanisms of immune checkpoint inhibitors (ICIs) and regulation of the tumor immune microenvironment (TIME). Tumor cells bind to PD-1 on the surface of T cells by overexpressing PD-L1 or PD-L2 molecules, thus inactivating T cells for immune escape. ICIs can inhibit these interactions by binding to PD-1 or PD-L1 and then activate cytotoxic T cells and other immune cells to kill tumor cells. **(A)**. TIL: Specific immune response to tumor cells. **(B)** APCs present antigens, stimulate T cells and transmit immune signals. **(C)** Th1: Mediates the cellular immune response and promotes cytotoxic T-cell (CTL) killing. **(D)** MDSCs: Inhibit the body immune cells to play normal innate and adaptive immune functions; Treg: Suppress the immune response of other cells and control self-tolerance) (by Figdraw).

As mentioned above, PD-1/PD-L1 blockades in the TIME exerts antitumor effects. On the one hand, as a part of the TIME, PD-1/PD-L1 blockades will change the composition or proportion of some immune cells in the TIME, but importantly, the composition of the TIME itself will induce the expression of PD-L1, affect the binding of PD-1/PD-L1 inhibitors and the subsequent recognition and phagocytosis of the immune response, and thus affect the antitumor effect (33). Existing studies have shown that the expression levels of target proteins, infiltrating T cells, and other types of immune cells in the TIME are closely related to the response to ICIs, which implies that the immune status of the TIME can determine the efficacy of ICIs (31). Studies have also shown that tumor-infiltrating lymphocytes (TILs) have potent and specific antitumor effects and are closely related to cancer prognosis (34–36). In particular, the effectiveness of immune checkpoint therapy can be predicted by CD8+ cells (34, 37). In addition, myeloid cells are heterogeneous immune cells of the innate immune system, represented by macrophages and dendritic cells (DCs), which have a powerful ability to modulate T-cell responses and play an important role in cancer progression. Therefore, any alterations in the TIME may affect the efficacy of ICIs; details are indicated in **Figure 2**.

### 3.2 Effects of COPD on the TIME in Patients with LC

Based on previous evidence, COPD has a significant impact on the TIME. We speculate that, on the one hand, the development of COPD is closely linked to the PD-1/PD-L1 pathway and the TIME and might be affected by various factors, such as T-cell apoptosis, altered expression of immune checkpoints on immune cells, and the effect of cytokines on immune cells or tumor cells. Some of the same pathogenesis might lead to a greater benefit of PD-1/L1 inhibitors (38, 39). On the other hand, the trend of COPD affecting some immune cells of the TIME might be consistent with PD-1/PD-L1 inhibitors or beneficial to PD-1/PD-L1 inhibitors to play a synergistic role or enhance the antitumor effects. Therefore, we discussed in detail how COPD affects the ICI efficacy of LC by modulating the TIME.

#### 3.2.1 Cluster of differentiation 4+ (CD4+) cells and T-helper cell type 1/2 (Th1/2)

In the airways and alveolar lumen of COPD patients, the number of CD4+ T cells increases significantly with airflow limitation and emphysema staging (40, 41). Upon encountering specific antigens, the initial CD4+ T cells will activate and differentiate into two effector T-cell subtypes to function. Th1 cells are the main effectors of phagocyte-mediated host immunity, which secrete interleukin 2 (IL-2), interferon γ (INF-γ) and TNF-α and are mainly involved in defense against intracellular pathogens. In contrast, Th2 cells secrete IL-4, IL-5, IL-6, IL-10 and other factors, which are involved in metabolic reactions and defense against parasitic infections (40).

Previous studies have indicated that Th1 cells secrete promyelocytic factors, which have antitumor effects, and Th2 cells secrete anti-inflammatory cytokines, which have tumor-promoting effects, and that the Th1/Th2 ratio is correlated with tumor stage (42). On the other hand, both Th1 and Th2 cells are increased in COPD and acute exacerbation of chronic obstructive pulmonary disease (AECOPD) compared to healthy individuals, and changes in the Th1/Th2 ratio correlate with the severity and prognosis of AECOPD (43). Thereby, we speculate that COPD affects tumor sensitivity to ICIs by affecting the ratio of Th1/Th2, and this hypothesis can be further confirmed by Mark et al. They confirmed that the number of CD8+ and CD4+ lymphocytes increased in COPD patients. Additionally, Th1 differentiation and PD-1 expression were increasingly affected by COPD lung tissue, which implied that the presence of COPD was associated with prolonged progression on-free survival in ICI-treated patients (19). An increased proportion of Th1 cells enhanced the antitumor immune response, and high levels of Th1 cells predicted better clinical outcomes after chemotherapy, while an increased proportion of Th2 cells downregulated the antitumor immune response and predicted worse chemotherapy outcomes (44). Therefore, CD4+ and Th1/2 cells play critical roles in the enhancement of ICI efficacy in LC with COPD.

#### 3.2.2 CD8+ cells and tumor-infiltrating lymphocytes (TILs)

CD8+ cells have become incredibly important in antitumor immunity research due to their direct antitumor cytotoxicity. In tumor immunity, CD8+ cells are activated upon recognizing tumor antigens presented on MHC-I, release IFN-γ to bind to its receptor, and induce the expression of PD-L1 on tumor cells, which could bind to elevated levels of PD-1 on the surface of TILs, thus triggering the inhibition of the PD-1/PD-L1 axis (23). ICIs enhance antitumor activity by blocking the interaction of PD-1 with PD-L1 and eliminating the suppressive effects of CD8+ cells. At the same time, TILs often become dysfunctional (‘exhausted’) and fail to destroy tumor cells due to prolonged exposure to persistent antigens or chronic inflammation. Surprisingly, clear evidence from earlier animal studies suggests that this phenomenon is reversible and that ICIs can restore the antitumor activity of TILs through PD-1/PD-L1 inhibitors, which in turn leads to a durable response in different subgroups of patients with solid tumors (24, 45, 46). Therefore, T-cell exhaustion is often considered a marker of tumor specificity and response to ICIs (45). Based on these two points, we hypothesized that the more PD-1/L1 expression or the stronger TIL exhaustion, the stronger the response to ICI may be.

By tissue analysis of NSCLC patients with and without COPD, Biton et al. revealed that COPD severity was positively correlated with CD8+ TIL depletion, as the expression of PD-1 and T-cell immunoglobulin domain and mucin domain-3 (TIM3) was enhanced in CD8+ cells from NSCLC patients with COPD (21). PD-1 and TIM3, as inhibitory receptors that induce T-cell depletion, are also considered markers of T-cell loss of function and tumor progression in NSCLC. Thus, it is evident that T-cell depletion mediated by its inhibitory receptors, such as PD-1, is present in patients with severe COPD. Furthermore, the results demonstrated that NSCLC patients with COPD had a higher survival rate after anti-PD1 therapy than those without COPD. Therefore, this study appears to highlight the effectiveness of PD-1 blockers in unleashing the antitumor CD8+ T-cell response in a subpopulation of patients characterized by strong CD8+ TIL depletion (21). This occurrence explains why COPD patients with fatigue but elevated TIL levels often respond better to ICI therapy than NSCLC patients without COPD.

#### 3.2.3 Regulatory T cells (Treg) and T-helper cell type 17 (Th17)

Treg and Th17 belong to the same T-cell subpopulation and are involved in the pathogenesis of CODP and LC. Th17 cells promote inflammatory responses, while Treg cells suppress them (47). There is an important balance between Th17 and Treg cells, which plays an important role in maintaining the immune environment, and an imbalance can lead to abnormal immune responses locally or systemically (40, 47).

Treg cells, a poor factor in cancer prognosis, can infiltrate tumors and suppress antitumor immunity within the TMIE, thus promoting tumor progression and growth (48). Th17 cells have complex biological functions, and evidence suggests that these cells may paradoxically also contribute to antitumor immunity (40). Under *in vitro* conditions, Th17 cells themselves cannot directly kill tumor cells but achieve antitumor immunity by stimulating tumor cells, promoting T-cell recruitment to tumor sites, and initiating CD8+ T-cell killing (40). Taken together, the development of COPD may alter the Treg/Th17 balance, which in turn affects the effect of immunotherapy on LC.

#### 3.2.4 Myeloid-derived suppressor cells (MDSCs)

MDSCs are a heterogeneous group of cells with significant immunosuppressive activity that promote tumor growth by suppressing effector T-cell function and thereby mediating tumor immune escape (47). Earlier studies indicated that in NSCLC, the synthesis of vascular endothelial growth factor (VEGF) was increased, and VEGF fostered the formation of MDSCs (49).

Meanwhile, Szentkereszty et al. determined that the effect of VEGF and MDSCs on systemic immunity was attenuated by the presence of COPD in patients with advanced NSCLC (50). They measured VEGF and MDSCs in patients with NSCLC or NSCLC combined with COPD. In NSCLC, a significant increase in VEGF and M-MDSCs and G-MDSCs was observed, whereas in NSCLC combined with COPD, M-MDSC scores were raised, yet G-MDSC scores remained constant. The study further analyzed the relationship between serum VEGF concentration and the size of various cell populations, again demonstrating a direct association between higher VEGF and M-MDSCs in NSCLC but the opposite relationship in NSCLC + COPD patients. Consequently, accompanying COPD decreased G-MDSCs and reversed the modulation of M-MDSCs by VEGF. The results suggested that PFS is positively influenced by COPD in advanced NSCLC because COPD supports some effector lymphatic function and alleviates tumor inflammation (50).

Overall, LC patients with COPD benefit more from ICIs due to their ability to alter the TIME. On the one hand, COPD-like chronic inflammation creates a favorable immunosuppressive TIME for tumorigenesis and development. On the other hand, COPD-related alterations in the TIME may lead to associated lung tumors overexpressing PD-L1 and PD-1, which respond better to ICIs.

## 4 New therapeutic strategies for LC with COPD

In current treatment guidelines, COPD is not an absolute contraindication of immunotherapy for LC patients but is considered a high-risk factor for the use of ICIs. The incidence of checkpoint inhibitor pneumonitis (CIP) in COPD and asthma patients was reported to be 2.3% higher than that in non-COPD or asthma patients (51) because LC patients with COPD require additional supervision after immunotherapy due to their weak lung function and immunity after long-term use of hormonal bronchodilators.

To achieve better therapeutic efficacy, some scholars have proposed identifying novel biomarkers or predictors to assess the risks or benefits of receiving ICIs. For example, Zhou et al. proposed that IL-2R may be used as a potential biomarker for ICI in patients with advanced LC and COPD, as high baseline levels of IL-2R and posttreatment elevations may predict poor prognosis (8). In addition, due to the existence of the same signaling pathways and pathogenic factors between COPD and LC, researchers have developed new drugs based on this idea, such as the development of new potential therapeutic targets using tobacco-related pathogenic mechanisms as an example (52). Studying the genome-wide association (GWA) of LC and COPD revealed identical single nucleotide polymorphisms (SNPs) in the CHRNA3-CHRNB4-CHRNA5 gene cluster (52), which provides a sound basis for the development of new drugs and therapeutic strategies (52). However, most studies have been limited to animal models or small sample clinical trials. In addition, antagonists of some cytokines have been proposed for use in combination with immunotherapy, such as IL-17 antagonists, which have been proposed for COPD treatment and LC chemoprevention because IL-17 cytokines are associated with cigarette smoke-induced emphysema, and inhibition of IL-17 limits disease progression (19, 53).

## 5 Discussion and summary

### 5.1 Summary

We performed a systematic evaluation and first reviewed the efficacy of PD-1/PD-L1 inhibitors during LC combined with COPD after immunotherapy and then analyzed the potential reasons for the benefit to the corresponding population, providing new evidence and viewpoints for clinical treatment. Our findings differ from previous findings that COPD, as an independent risk factor for LC, is generally considered detrimental to patient treatment and prognosis. In contrast, we concluded that LC patients with COPD benefited better from anti-PD-1/PD-L1 therapy, with improved lung function, such as FeNO levels, FEV1, and FVC, and prolonged OS, PFS and ORR. The reason for these benefits may be that COPD alters the TIME of the LC, mainly manifested by increased Th1 expression, increased PD-1 expression on CD8+ cells, enhanced TIL exhaustion, an altered Treg/Th17 ratio, reduced G-MDSCs and reversed effects of VEGF on M-MDSCs.

### 5.2 Other potential mechanisms by which LC patients with COPD benefit from ICIs

In addition to changes in the TIME, COPD and LC have also been found to have altered epigenetic modifications, including DNA methylation and microRNA regulation (47–49). On the one hand, DNA methylation regulators have long been considered potential biomarkers for assessing the efficacy of ICIs (54), and studies have demonstrated that DNA methylation profiles can effectively infer the proportion of different types of immune cells in the TIME (55), and promoter methylation levels of CTLA4, LAG3, and PD-L1 are associated with efficacy-related immunotherapy (56). On the other hand, several studies have revealed that DNA methylation contributes to COPD development and serves as a potential biomarker for COPD disease prevention, diagnosis and prognostic assessment (57, 58). Therefore, we speculate that the modulation of DNA methylation in COPD and LC affects the efficacy of ICIs. For example, Wauters et al. analysed COPD-driven immune-related signatures by DNA methylation profiling of NSCLC and revealed some differences between LC patients with and without COPD, namely, the different levels of expression and methylation of genes that are primarily involved in the immune response (59). Recently, an epigenome-wide association study (EWAS) carried out the link between the gene methylation of COPD and LC. According to the methylation level and the degree of gene repression, COPD+LC was the highest, COPD was the second, and LC was the lowest (60).

MicroRNAs are short, single-stranded RNAs that play important roles in the pathophysiological processes of many diseases. Previously, some commonly dysregulated microRNAs have been identified in both COPD and LC and can be utilized as novel therapeutic targets, as well as for early diagnosis and prognosis (61). Fathinavid A et al. revealed that miRNA targets such as hsa-miR-15b and hsa-miR-106a are associated with COPD and LC, which are downregulated in COPD but upregulated in NSCLC (61). Yang et al. (62) also stated that the expression level of miR-103 was downregulated in NSCLC and COPD tissues, while it was inversely correlated with tumor stage and tumor size. Meanwhile, miR-106a is an oncogenic miRNA that targets the transcription factor FOXO3, thereby regulating apoptosis, cell cycle arrest, and autophagy-related genes (61, 62). We speculate that unlike LC or COPD alone, microRNAs are dysregulated in LC with COPD, thereby affecting the efficacy of ICIs.

Whether it is the alteration of the TIME or epigenetic (DNA methylation/MicroRNAs), more detailed studies on the molecular mechanism of lung cancer combined with COPD, such as the study of SNPs, exploration of biomarkers, and epigenetic modulation, are needed in the future to provide more basis for the clinical application of ICIs for lung cancer combined with COPD.

### 5.3 Safety for LC patients with COPD receiving ICIs

Although LC patients with COPD benefit from ICIs, some side effects occur during treatment, such as ICI-related pneumonia (IRP), thyroid toxicity, and dermal toxicity. In particular, IRP is very typical and closely related to the use of ICI in inflammatory situations. IRP is an inflammatory and invasive lung disease associated with ICI, which has a high termination rate and mortality, leading to discontinuation of treatment in LC patients (63, 64). Previous pairwise meta-analyses reported that the incidence of IRP in NSCLC was 3.6-4.1%, and the incidence of IRP in PD-1 inhibitors was higher than that in PD-L1 inhibitors (64, 65). Moreover, COPD, as an inflammatory disease, is one of the potential risk factors for IRP. Therefore, both the efficacy and safety of ICIs should be carefully considered during the treatment of LC patients with COPD. For IRP that has occurred, international organizations or guidelines recommend that CIP be divided into different types and levels according to imaging and clinical symptoms. The treatment principles are as follows: appropriately delay ICI treatment + symptomatic treatment such as hormone + follow-up (mild IRP, level 1), suspend ICI treatment + symptomatic treatment such as hormone + hospitalization (moderate IRP, level 2), permanently stop ICI + symptomatic treatment such as hormone + hospitalization (severe IRP, level ≥3) (66). Then, for patients without IRP temporarily, we suggest establishing some predictive biomarkers or prediction models to avoid ADR. Some studies have built a prediction model for IRP and used COPD as one of the predictors to judge the probability of IRP occurrence by scoring patients’ COPD and other factors (physical fitness score, ages) to provide treatment decisions for the clinical selection of ICI (63).

### 5.4 Advances in detection of PD-L1 expression

PD-L1 expression is a commonly used biomarker to predict ICI efficacy, and we attempted to find the optimal cut-off value of immunotherapy for LC patients with COPD, but there were no data available on the relationship between the expression level of PD-L1 and the efficacy of immunotherapy. We systematically evaluated the LC group and COPD+LC group, both including PD-L1<1%, 1-49% and ≥ 50%. However, there was no significant difference between the two groups. Therefore, research on the relationship between PD-L1 expression and immunotherapy efficacy may be a novel direction of future research.

Currently, several methods are available to detect the expression of PD-L1. 1) Immunohistochemistry (IHC): IHC staining is a classic method to detect protein expression, and the NCCN guidelines recommend IHC detection of PD-L1 expression in NSCLC. The FDA has also approved a variety of PD-L1 IHC tests as a concomitant diagnosis of whether tumor patients should receive ICIs (67). 2) Multiplex fluorescence (mIF): mIF is a promising tool in the scenario of immunotherapy because it can simultaneously detect and quantify PD-L1 markers with multiple antibody clones and conduct in-depth analysis of the number, density and spatial location of tumor and immune cells. MIF has been widely used at present, and it is expected to be a powerful clinical tool for accurate prognosis and prediction of efficacy, to help accurate prediction of efficacy, and to facilitate accurate screening of patients benefiting from immunotherapy (68, 69). 3) Imaging Mass Cytometry (IMC): This method can simultaneously analyze more than 40 markers on a single tissue slice. More importantly, it can realize the *in situ* detection of protein expression and ensure the integrity and accuracy of data. Especially for some precious microsamples, invalid loss during sample preparation is avoided (70). Previously, Alnajar et al. studied the immune cell repertoire and PD-L1 expression in patients with sarcomatoid urothelial carcinoma (SUC) using IMC, which promoted the understanding of the rare subtype of urothelial carcinoma (UC) (71). 4) Others: In addition to PD-L1, a growing body of data suggests the importance of immune cells and other important biomarkers in the TIME in guiding patients’ drug selection and prognostic assessment (31). For example, circulating tumor cells (CTCs) can be detected and PD-L1 expression can be dynamically evaluated in a timely manner to identify drug resistance to the current treatment scheme and guide follow-up treatment. Immunoosmosis detection methods are also emerging, such as high-precision single-cell RNA sequencing and bulk RNA-seq.

### 5.5 Future perspective

The current review has some limitations: (1) There are few studies related to immunotherapy in LC patients with COPD, and more high-quality prospective studies are needed to verify these conclusions. (2) Most LC patients with COPD are over 60 years old, and the risk of adverse reactions is high, while we did not report the safety outcome. (3) Evidence related to the effects of SCLC and long-acting bronchodilator data on the efficacy of ICIs is lacking. (4) This study did not investigate the effects of CTLA-4 inhibitors on the efficacy in LC patients with COPD.

In general, the following areas should be considered in future research. First, from the perspective of the mechanisms for better efficacy of ICIs in LC patients with COPD, further studies on both the alteration of the TIME and epigenetic regulation (such as DNA methylation and noncoding RNA modulation) are of great importance. Moreover, research on microenvironment-related and immune-related gene SNPs and exploration of biomarkers for predicting the efficacy of ICIs in LC patients may provide a basis for the clinical application of ICIs in the treatment of lung cancer combined with COPD. Second, we should pay attention to the safety of ICI treatment in the COPD+LC population, especially the screening criteria for the expression level of PD-L1 or other biomarkers for ICI-related ADRs, and establish prediction models for IRPs. Last, at present, the monitoring of immunotherapeutic markers is mainly aimed at the tumor itself, but there is no objective monitoring and evaluation of the entire TIME. Therefore, we believe that the TIME should be monitored in the future, including TILs and Tregs, and new biomarkers related to the TIME should be explored.

## 6 Conclusion

In short, LC patients with COPD could benefit from anti-PD-1/PD-L1 therapy, which may be due to changes in the TIME (an altered Th1/2 ratio and Treg/Th17 ratio, increased consumption of CD8+ cells and TIL and MDSC reversal), ultimately improving lung function and prolonging the OS or PFS of patients. In the future, research on the relationship between the expression level of PD-L1 and the efficacy of immunotherapy, as well as the detailed molecular mechanisms by which COPD affects the TIME and ICI efficacy, would provide a novel basis for ICI treatment in LC patients with COPD.

## Author contributions

ML and ZH conceived and designed the review. ML wrote the manuscript. YC prepared the figures. HX, ZH, and TW reviewed and edited the manuscript. All authors read and approved the final manuscript.

## Funding

This work was mainly supported by the Sichuan Science and Technology Program (2021YFH0144 and 22ZYZYTS0152).

## Conflict of interest

The authors declare that the research was conducted in the absence of any commercial or financial relationships that could be construed as a potential conflict of interest.

## Publisher’s note

All claims expressed in this article are solely those of the authors and do not necessarily represent those of their affiliated organizations, or those of the publisher, the editors and the reviewers. Any product that may be evaluated in this article, or claim that may be made by its manufacturer, is not guaranteed or endorsed by the publisher.

## Abbreviations

LC, Lung cancer; COPD, Chronic obstructive pulmonary disease; AECOPD, Acute exacerbation of chronic obstructive pulmonary disease; ICIs, Immune checkpoint inhibitors; PD-(L)1, Programmed death-1/programmed death-ligand 1 inhibitors; NSCLC, Non-small cell lung cancer; TIME, Tumor immune microenvironment; CD, Cluster of differentiation; DCs, Dendritic cells; TILs, Tumor-infiltrating lymphocytes; Th1/2, T-helper cell type 1/2; Treg, Regulatory T cells; MDSC-s, Myeloid-derived suppressor cells; IL-2, Interleukin 2; TNF-γ/α, Tumor necrosis factor γ/α; TIM3, T cell immunoglobulin domain and mucin domain-3; MDSCs, Myeloid-derived suppressor cells; VEGF, Vascular endothelial growth factor; EWAS, Epigenome wide association study; OS, Overall survival; PFS, Progression-free survival; ORR, Objective response rate; FEV1, Forced expiratory volume in one second; FVC, Forced vital capacity; IRP, ICI-related pneumonia; APCs, Antigen presenting cells; ADR, Adverse drug reactions; MHC, Major histocompatibility complex; Mif, Multiplex fluorescence.
